# Supplementary material for: Can Health-Enhancing Sporting Programs in Sports Clubs Lead to a Settings-Based Approach? An Exploratory Qualitative Study
Source: Int J Environ Res Public Health. 2021 Jun 4;18(11):6082. doi: 10.3390/ijerph18116082 (PMC8200194; doi:10.3390/ijerph18116082)
Supplement: Supplementary file 1 [file ijerph-18-06082-s001.zip › File S1_interview guide sports clubs.pdf]

Supplemental file 1: Interview guide sports clubs

Date of interview:
Sporting program:
Sports club:
Number of members of sports club:

Background information respondent:
Name:
Function within sports club:
Number of years employed with the sports club:
Involved in the sporting program since (year):
Gender:
Age:

1. Implementation/progress program:

- When did the sports club start providing <<name sporting program>>?
- What happened with <<name sporting program>> in the years thereafter (important moments/changes)?
- What are the most important reasons for the sports club to continue offering <<name sporting program>>?

2. Program design:

- How does the sports club provide <<name sporting program>> (type of activities, duration and frequency of training sessions, group size, etc.)? Did (the content of) the sporting program change since the start?
    - If yes, what has changed, when and why?
    - If no, why not?
- Wat is the current target group of <<name sporting program>>?
    - <<name sporting program>> was part of the National Action Plan for Sport and Exercise (NAPSE). Within the NAPSE, the focus was on inactive target groups. Are inactive people still (part of) the target group? Please explain why they are or are not (part of) the target group (anymore).
    - Are you able to reach/include inactive people with <<name sporting program>>?
        - If yes, how do you reach/include inactive people?
        - If no, why not?
- How often does the sports club provide the activities per year?
- How many participants are there (on average) per activity per year?
- How many participants become (on average) member of the sports club due to participation in <<name sporting program>>?

3. Organizational setting sports club:

- How is <<name sporting program>> organized/run within the sports club (who is involved, what are their roles and tasks)? Did the organization of the sporting program change during the years? If yes, how and why?
- Does the National Sports Federation still provide support to the sports club to run <<name sporting program>>?
    - If yes, how?
    - If no, why not?

- Does the sporting program fit with the sports club’s tasks and aims? If yes/no, please explain.
- Is there (sufficient) support for the sporting program within the sports club? If yes/no, please explain.
- Is the sporting program part of the sports club’s long-term policy?
- Is implementation of <<name sporting program>> by the sports club financially secured (e.g., with sponsorship, participant fees)? If yes/no, please explain.

4. **Trainer/coach:**

- Did the sports club recruit new trainers and/or are trainers (within the club) educated to run <<name sporting program>>? And if yes, are they still available to the club?
- Are there sufficient (educated) trainers?
- Are there possibilities to educate new trainers and/or are there extra training options (refreshment courses) for current trainers?

5. **Broader community environment:**

- Did the sports club collaborate with other people/organizations to run <<name sporting program>>?
  - If yes, with whom and how?
  - Does the sports club still collaborate with these people/organizations?
  - Are partnerships for <<name sporting program>> stopped? If yes, with whom and why?
- Is there (sufficient) support for the sporting program outside the sports club (e.g., with the target group, in the community, with partners)? If yes/no, please explain.

6. **Results/effects of sporting program:**

- What are the most important results of <<name sporting program>> for the sports club?
- Is <<name sporting program>> of added value to the sports club in comparison with already existing/regular activities?
- What are the most important results/effects of <<name sporting program>> with participants? Do they enjoy participating in the program?
- Do participants of <<name sporting program>> keep participating in the sport (inside/outside the club)? And how do you encourage this?

7. **Facilitating and impeding factors for continuation of the sporting program:**
   When considering all that is previously discussed:

- What are the most important factors that facilitated/supported continuation of <<name sporting program>> by your sports club?
- Are there any factors that impeded continuation of <<name sporting program>> by your sports club?

8. **Future of sporting program:**

- Does your sports club intend to provide <<name sporting program>> in the future?
  - If yes, how?
  - If no, why not? Is the sporting program replaced by other activities?
- Are there any challenges/barriers for your sports club to continue the sporting program in the future?

9. **Remaining remarks:**

- Do you have anything to add to this interview? For example, something that is important for (continuance of) <<name sporting program>>, but what we did not discuss before.
