# Supplementary material for: Can Health-Enhancing Sporting Programs in Sports Clubs Lead to a Settings-Based Approach? An Exploratory Qualitative Study
Source: Int J Environ Res Public Health. 2021 Jun 4;18(11):6082. doi: 10.3390/ijerph18116082 (PMC8200194; doi:10.3390/ijerph18116082)
Supplement: Supplementary file 1 [file ijerph-18-06082-s001.zip › File S2_activities per level of the sports club.pdf]

Supplemental file 2: Description of activities per level (micro-, meso-, macro-level) of the sports club

Table. Description of activities per level (micro-, meso-, macro-level) of the sports club (n=16)

Sports club	Micro-level (activities trainer)	Meso-level (activities leading persons in the club)	Macro-level (policy activities within the club and activities concerning the broader community environment)
1	Three certified running trainers are involved in the running program.<br>• They provide running and social activities to participants.<br>• They participate in educational activities.<br>• One trainer coordinates the program.		• The running program is part of the sports club's policy (year planning).<br>• The sports club collaborates with a running store to promote the running program.<br>• The NSF facilitates the sports club with (promotional) materials, education possibilities and financial resources.
2	Three certified judo trainers are involved in the judo program.<br>• They educate neighborhood sport coaches to provide the judo lessons in schools.<br>• They provide follow-up activities at the sports club.<br>• They participate in educational activities.	Two board members are involved in the judo program.<br>• They bring judo mats and suits to the schools. They place the mats in the schools and clean-up afterwards, including cleaning the mats and washing the suits.<br>• One board member (the secretary) coordinates the program.	• The judo program is part of the sports club's long-term policy.<br>• The sports club is part of a platform which promotes physical activity among youth and in which the sports club collaborates with two municipalities, eight neighborhood sport coaches who provide the judo lessons in schools, schools, and another sports club.<br>• The municipality facilitates the sports club with financial resources (i.e., for travelling-expenses, washing/cleaning, the train-the-trainer courses and hiring the judo mats and suits).<br>• The NSF provides the judo mats and suits to the club against payment and facilitates the sports club with education possibilities.
3	There are three certified walking trainers (including the head trainer) and eight certified walking guides involved in the walking program.<br>• Trainers provide the walking (club's own walking program next to the individual Through 4 Days Marches program) and social activities to participants.<br>• The guides assist the walking trainers during the walking and social activities.<br>• Trainers provide informational meetings to participants about the Four Day March in Nijmegen and their training schedule.	• The chairman, who is also head trainer, coordinates the program.	• The walking program is part of the sports club's long-term policy (the main aim of the sports club is to prepare people to walk the Four Day March in Nijmegen).<br>• There are seven sponsors that support the sports club in the form of financial resources (so that participation costs for the program and social activities can be kept low) and materials (T-shirts, bags, and other materials for participants).<br>• The NSF facilitates the sports club with education possibilities, financial resources (fee for organizing the informational meetings) and provides content for the informational meetings.

	• Trainers and guides participate in educational activities and trainers provide educational activities (train-the trainer (renewal) courses) to other trainers and guides.		
4	There are four certified gymnastics trainers (including the head trainer) involved in the gymnastics program.<br>• They provide gymnastics and social activities to participants.	• The head trainer, who is also owner of the gym (this is a commercial sports club), coordinates the program.	• The sports club collaborates with the municipality which provides financial resources for providing the program (i.e., the prevention of falls program for (inactive) older adults with physical limitations).<br>• There is collaboration with a physiotherapist who provides the prevention of falls program with the head trainer and promotes the program among (inactive) older adults with physical limitations.
5	There is one trainer (this person has no official trainer's diploma) involved in the gymnastics program.<br>• This trainer provides the gymnastics activities to the participants.		
6	There is one trainer (this person has no official trainer's diploma) that is involved in the hockey program. This person is also participant in the program.<br>• This trainer provides the hockey and social activities to the participants, but other participants can lead the hockey and social activities as well.<br>• This trainer coordinates the program.		• The hockey program is part of the sports club's long-term policy.<br>• The sports club collaborates with the senior's club. New participants are recruited through the senior's club (or by word of mouth) and the senior's club provides financial resources for their club members to the hockey club so that participation fees can be kept low (most (n=25) participants in the program are also member of the senior's club).
7	There is one trainer (this person has no official trainer's diploma but is movement scientist and former teacher) that is involved in the hockey program.<br>• This trainer provides the hockey and social activities to the participants.<br>• This trainer coordinates the program.		
8	There are two certified bridge teachers involved in the bridge program.<br>• They provide the bridge and social activities to participants.		• There is collaboration with the municipality (on a project basis) who finances the program (so that participation fees can be kept low) and helps in recruiting the target group (the municipality sends an invitational letter to participate in the program to all older adults (60+ years) in the municipality).

			• The NSF recruits municipalities, coordinates the program at the local level, provides financial resources to pay the bridge teachers and supports the bridge teachers with (bridge) materials.
9	There are three certified bridge teachers (including the board member) and multiple table guides (club members, volunteers) involved in the bridge program.<br>• The bridge teachers provide the bridge and social activities to participants.<br>• Two or three table guides assist the bride teacher during each lesson.	• One board member, who is also bridge teacher, coordinates the program within the sports club.	• There is collaboration with the municipality (on a project basis) who finances the program (so that participation fees can be kept low) and helps in recruiting the target group (the municipality sends an invitational letter to participate in the program to all older adults (55+ years or 65+ years) in the municipality).<br>• There is collaboration with a sport service organization to provide physical activities to participants.<br>• The NSF recruits municipalities, coordinates the program at the local level, provides financial resources to pay the bridge teachers and supports the bridge teachers with (bridge) materials.
10	There are certified cycling trainers from the education committee involved in the cycling program.<br>• They provide the cycling and social activities to participants.<br>• They participate in educational activities and provide educational activities (train-the-trainer courses) to other (new) trainers.	There is an education committee consisting of certified trainers.<br>• This committee coordinates the program.<br>• This committee facilitates education possibilities for (new) trainers.	• The cycling program is part of the sports club's long-term policy.<br>• The sports club collaborates with the neighborhood association and a mountain bike shop to promote the program.<br>• The NSF facilitates the sports club with (promotional) materials, education possibilities and financial resources.
11	There is one certified cycling trainer and there are six club volunteers involved in the cycling program.<br>• The trainer coordinates the program and provides the cycling activities to participants.<br>• The volunteers provide the cycling activities and/or assist the trainer in guiding participants during the training.<br>• The trainer participates in educational activities.		• The sports club collaborates with local merchants and health organizations to promote the program.<br>• The NSF facilitates the sports club with (promotional) materials, education possibilities and financial resources.
12	There are two guides (the guides have no trainer diploma) involved in the cycling program. The guides are also participants in the cycling program.<br>• They coordinate the program (they choose the cycling routes and social activities).		

	• They guide the other participants during the cycling and social activities.		
13	There are six guides (two guides per group; the guides are volunteers of the club and have no trainer diploma) involved in the cycling program.<br>• They guide the participants during the cycling and social activities.	There is a committee initiated specifically for the cycling program consisting of five members.<br>• The committee coordinates the program.<br>• Each committee member has his or her own task: a member is chairman, a member chooses the cycling routes, a member hosts the activities, a member selects volunteers that guide participants during the cycling activities and a member does administrative tasks.	• The cycling program is part of the sports club's long-term policy.
14	There are multiple volunteers involved in the Trio-Triathlon event.<br>• These volunteers organize the event.		
15	There are two certified volleyball trainers and different volunteers of the club involved in the volleyball program.<br>• They provide the volleyball and social activities to participants.<br>• One trainer coordinates the activities.		• The program is part of the sports club's long-term policy (CMV is the official volleyball form to teach children 6 to 12 years the fundamentals of volleyball at volleyball clubs and the official competition form for this age group).<br>• The sports club collaborates with other local clubs to organize CMV tournaments.
16	There are three certified volleyball trainers involved in the volleyball program.<br>• The trainers provide the volleyball activities to the participants, sometimes supported by volunteers (parents, other club members).<br>• Trainers provide educational activities (train-the-trainer courses) to new trainers.<br>• One trainer coordinates the activities.		• The program is part of the sports club's long-term policy (CMV is the official volleyball form to teach children 6 to 12 years the fundamentals of volleyball at volleyball clubs and the official competition form for this age group).
